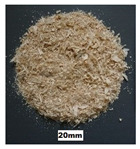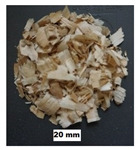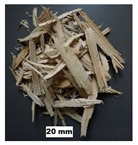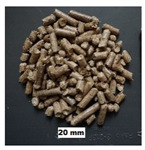# Friction and Shear Properties of Pine Biomass and Pellets

**DOI:** 10.3390/ma13163567

**Published:** 2020-08-12

**Authors:** Mateusz Stasiak, Marek Molenda, Maciej Bańda, Józef Horabik, Joanna Wiącek, Piotr Parafiniuk, Justyna Wajs, Marek Gancarz, Ewa Gondek, Aleksander Lisowski, Tomasz Oniszczuk

**Affiliations:** 1 Institute of Agrophysics, Polish Academy of Sciences, Doświadczalna 4, 20-290 Lublin, Poland; m.molenda@ipan.lublin.pl (M.M.); m.banda@ipan.lublin.pl (M.B.); j.horabik@ipan.lublin.pl (J.H.); j.wiacek@ipan.lublin.pl (J.W.); p.parafiniuk@ipan.lublin.pl (P.P.); j.wajs@ipan.lublin.pl (J.W.); m.gancarz@ipan.lublin.pl (M.G.); 2 Department of Food Engineering and Process Management, Institute of Food Science, Warsaw University of Life Sciences, Nowoursynowska 166, 02-787 Warsaw, Poland; ewa_gondek@sggw.edu.pl; 3 Department of Biosystems Engineering, Institute of Mechanical Engineering, Warsaw University of Life Sciences, Nowoursynowska 166, 02-787 Warsaw, Poland; aleksander_lisowski@sggw.edu.pl; 4 Department of Thermal Technology and Food Process Engineering, University of Life Sciences in Lublin, Głęboka 31, 20-612 Lublin, Poland; tomasz.oniszczuk@up.lublin.pl

**Keywords:** granular biomass, friction, strength, torque

## Abstract

Knowledge on the mechanical properties of granular biomass is important for the design and efficient operation of equipment used for handling, storage, and processing. Their mechanical properties are used as a measure of material quality. In this study, the mechanical properties of granular biomass obtained from pines (sawdust, shavings, long shavings, and pellets) were determined under a moisture content range of 10–50%. The coefficient of sliding friction µ of four construction materials was determined using a 210-mm-diameter direct shear tester (Jenike’s shear box). To measure the shear resistance of the biomass materials (represented as torque T), a prototype vane tester was constructed. The characteristics of shear resistance with respect to time T(t) were determined for material samples under normal pressure *p* ranging from 5 to 30 kPa and a vane rotation rate of 3 rpm. Measurements were performed for five geometries of the rotor, reflecting typical deformation conditions encountered in the processing of granular biomass. The coefficient of sliding friction was found to be affected by the type of material, moisture content, and normal compressive pressure. Depending on the biomass material, the highest µ, which ranged from 0.50 to 0.62, was obtained for black steel, whereas the lowest µ, which ranged from 0.27 to 0.52, was obtained for aluminum. The lowest coefficient of sliding friction was observed for dry materials and high normal pressure. The torque T was observed to be affected by the rotor shape, material, normal pressure, and moisture content. The parameters presented provide information useful for the design of transport equipment and processing of granular wood biomass.

## 1. Introduction

The share of biomass used in firing and co-firing processes is constantly increasing. Hence, knowledge on the behavior of these materials during dosage processing and their reaction with construction materials is necessary [1,2,3,4,5,6,7,8]. Biomass is usually used in the granular form, which may generate problems during technological processes, including flow stops, caking during storage, disruption of dosing continuity, and self-segregation. Granular biomass materials used for power generation take the form of sawdust, woodchips, and pellets. Technological equipment used for their processing should be properly designed to ensure optimum conditions. Pine tree is one of the most popular and widely available sources of biomass worldwide. Pine biomass in the form of woodchips, shavings/sawdust or densified as briquettes or pellets is widely used as a renewable source of energy. Characteristics such as density and parameters of strength and friction are of particular interest in the bioenergy market. Those strength properties standardized in design codes (such as [9]) are necessary for the design of storage facilities and handling appliances. The parameters allow for estimates of the pressures exerted on storage structures by granular materials and ensure reliable flow. In the majority of cases, methods for determining the mechanical properties of biomass are adopted from earlier contributions in the fields of geotechnics and soil mechanics. Usually, these standard methods and apparatuses require resizing and redesign to properly treat larger biomass particles.

Currently, the methods used for the determination of granular biomass parameters are still under development. Several methods have been used to determine the parameters and characteristics in soil mechanics and granular food mechanics. A detailed description of these methods was presented by Stasiak et al. [10]. The mechanical properties of granular biomass were found to be strongly influenced by the particle size distribution and moisture content [7], and granular biomass has been studied by many researchers [3,8,11,12,13,14,15,16].

Moreover, ongoing efforts have been made to build and implement equipment for the testing of granular biomass. These new methods and equipment have been described by Stasiak et al. [17]. However, users of granular biomass require quick and easy testers to determine the flowability, strength, density, and moisture content of the granular biomass. The properties of biomass have been investigated for several years, but no standard procedures have been established for the determination of the mechanical properties of granular biomass [10,17]. There are also no data in the literature regarding the influence of the shape of the rotor on the torque generated in consolidated granular biomass. There is still a lack of data on the coefficient of sliding friction between biomass, especially pine biomass materials and construction materials at different moisture contents. The parameters determined in this study can be helpful in the design of equipment for the processing and transport of granular biomass.

In the present project, measurements were conducted to determine the coefficient of sliding friction of construction materials and the torque resistance of rotating vanes in compacted granular biomass using a direct shear tester and newly constructed vane shear tester, respectively. This study aims to determine (a) the coefficients of sliding friction between different biomass and construction materials and (b) torques opposing the movement of the rotor in consolidated granular biomass. Torque measurements were performed using a new device based on the equipment developed by Stasiak et al. [17]. The principal modification of the previous device was downsizing to allow easier mobility and field measurements. The new apparatus enabled a simple measurement of the density of biomass, which could be useful for practitioners. The density of granular materials is a very important parameter for numerous practical applications. It exerts a significant effect on the mechanical characteristics of the material and is used for the estimation of the pressure exerted by the granular material against the structure of the bin or silo. The effect of the rotor shape on the torque and density of materials was also examined.

A significant amount of literature data relates to biomass obtained from different types of sources. There is lack of datum concerning the mechanical characteristics of the different types of biomass obtained from pine tree. No summarized paper on the mechanical parameters of pine biomass of different levels of fragmentation is available and our paper is a contribution to the knowledge.

## 2. Materials

In this study, four materials of pine origin, i.e., sawdust, shavings, long shavings, and pellets were examined. The same materials were selected by the authors in their previous study on flowability and strength of the same materials [18]. Sawdust and shavings I and II, with different particle sizes, were obtained from a local factory, whereas pine pellets were obtained from a local pellet producer. Images of the tested biomass materials and particle size distributions of the tested materials are shown in Table 1. After the delivery of the materials, sawdust and shavings were dried in thin layers under laboratory conditions. Then, distilled water was added, and the materials were mixed for 15 min every hour for 24 h in a laboratory mixer to obtain the desired moisture contents w: 10%, 20%, 30%, 40%, and 50%. The levels of moisture content corresponded to those existing in technological practice, wherein biomass under various conditions is delivered, depending on the place of origin, season, and weather. The levels of moisture content are comparable to those used in other studies [15,19]. In the case of pellets, the influence of moisture content was not examined. The moisture content was measured by weighing 200–300 g samples before and after 24 h of drying at 105 °C in a laboratory oven. The particle size distributions of the sawdust and woodchips are presented in Table 1. In the case of sawdust, a sieve analysis was performed, whereas for woodchips, the length of the particles was measured with a digital caliper. In both cases, 0.5 kg of material was examined.

Aluminum, black steel, stainless steel, and galvanized steel sheets were used in the direct shear test and to determine the coefficient of sliding friction.

## 3. Methods

The coefficient of sliding friction µ between the biomass samples and sheets of construction materials were determined using a direct shear tester, with a chamber diameter of 210 mm. The test was performed based the Eurocode 1 [9] standard with normal pressure p of 15 and 30 kPa and shearing speed of 0.17 mm·s^−1^. The normal pressure used in the tests corresponded to the loads in storage silos approximately 10 m high, which are used in energy plants where the continuous energy production must be maintained through co-firing with standard coal fuel. Based on the experimental force–displacement curves, the coefficient of sliding friction between the biomass materials and construction materials was determined. The coefficient is calculated by dividing the shear force and the vertical load applied to the sample in the direct shear apparatus as recommended by [9].

**Figure 1:**
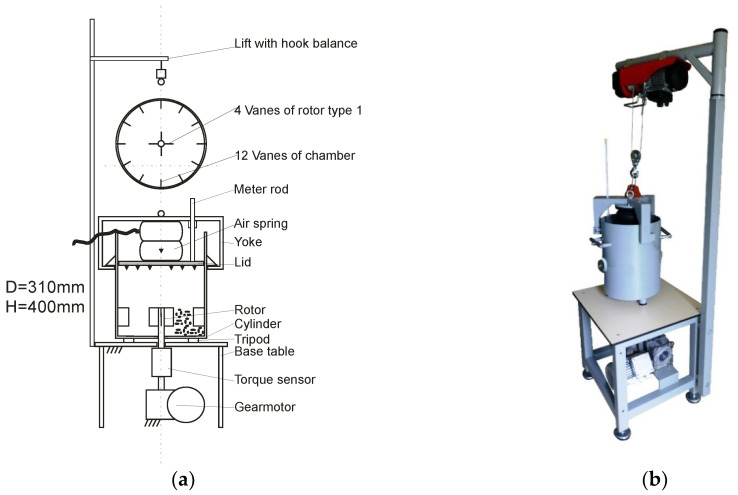
Vane shear tester: schematic of the apparatus with rotor type1 (**a**) and measuring station with lift and balance (**b**).

Torque T measurements in the consolidated biomass samples were performed in a vane tester. The apparatus previously used by Stasiak et al. [17] for determining the shear behavior of woodchips and pine biomass was modified for the purpose of this project, and a simpler vane shear tester was used. Schematic views of the tester and measuring station are presented in Figure 1.

**Figure 2:**
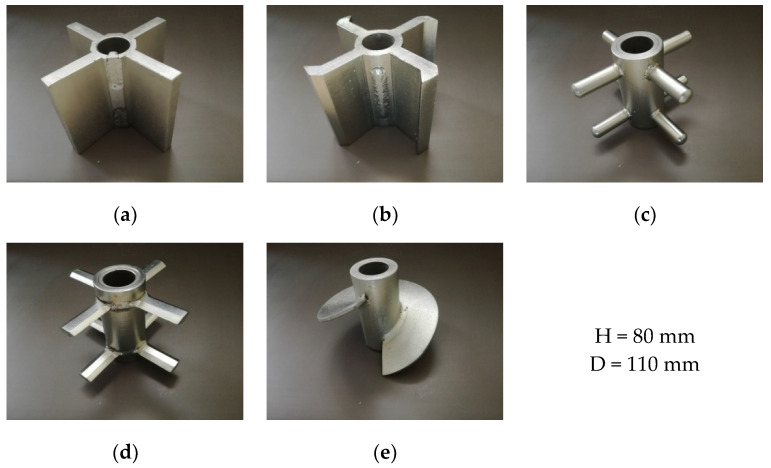
Rotors used in experiments. (**a**) Vane rotor, (**b**) sharp vane rotor, (**c**) rod rotor, (**d**) knife rotor, (**e**) screw rotor.

The basic part of the tester is a cylindrical chamber, with a diameter of 310 mm and height of 400 mm. Inside the chamber, axially, near the bottom, a rotating rotor measuring 8 cm high and 11 cm wide and featuring four blades are located. The five different rotors presented in Figure 2 are impelled by a gear motor with an adjustable rotation rate. The geometry of the rotors is similar to that used in silo unloaders, mechanical bin actuators, and arch breakers.

Normal pressure was exerted by the pneumatic system with a rubber air spring and yoke. The rotating vane impeller was assumed to shear only the material placed in the immediate vicinity of the blades. Twelve blades with dimensions equal to those of the blades of the rotor were installed on the internal surface of the chamber. The test chamber was supported by the base table and connected with the drive by a claw clutch. The concept of the proposed device is based on a combination of the rheometer and the new system of vertical loading with the yoke and rubber air spring filled with air to the required pressure p. The mass of the sample was measured with a hook weight balanced with a rotor and chamber. This was in contrast to the previous version where three load cells were used to support the chamber. The actual height of the sample was measured with a meter rod. With such equipment, the volume, mass and density of materials can be measured. Density ρ was determined before shearing and after filling and compressing the material inside. The weights of the chamber and rotors were not accounted in determining the density.

A torque sensor was used to measure the shear load on the rotating rotors. The tests were conducted with a rotation rate of 3 rpm and four magnitudes of normal pressure: 5, 10, 20, and 30 kPa. The required normal pressure was generated by a compressor and measured by analogue and digital manometers. The relationship of torque with time T(t) and other measured parameters were recorded by a data acquisition system. The test procedure included filling of the chamber, application of normal pressure and shearing with constant speed. After the maximum torque Tmax1 was attained, the gear motor was stopped. After a rest period of ~50 s, when the torque decreased to an asymptotic value, second shearing was performed to attain the maximum value Tmax2. Subsequently, the sample was unloaded, and the chamber was emptied. The vane shear tester was also used to determine the density in relation to the normal pressure. Density was measured after free-filling the chamber with a rotor inside the cylinder.

All the experiments were performed thrice. The analysis of variance of the obtained data was performed using Statistica (version 12, StatSoft, Cracow, Poland) [20]. Information on the statistical significance was given as 0.95 confidence intervals shown as bars on the drawings. p is the calculated probability (or level of statistical significance; p < 0.05 means statistically significant), and F is the value of Snedecor’s test function. The higher the value of F, the stronger the effect of a given factor on a determined parameter. The numbers in brackets at F indicate file variables analyzed by software. In each of the graphs they are different because other variable factors are taken into account.

## 4. Results

### 4.1. Bulk Density

**Figure 3:**
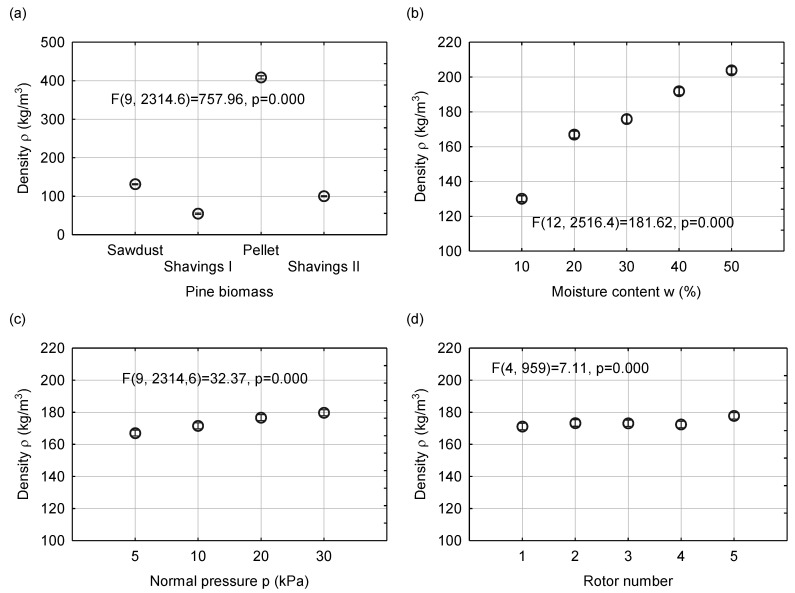
Mean value with 0.95 confidence intervals of bulk density calculated for the experimental materials (**a**) and mean bulk density influenced by the moisture content (**b**), normal pressure (**c**), and rotor number (**d**).

In this study, the density of material was determined, that is, the most important parameter of granular biomass that allows to project the capacity of the silos for a given material. The density was measured with a rotor inside the cylindrical chamber of the industrial vane shear tester. The densities measured after filling the chamber with pine biomass materials and the relationships between the density averaged for different material types, moisture contents, normal pressures, and type of rotor are presented in Figure 3.

Among the experimental materials, the highest mean bulk density of 400 kg/m^3^ was obtained for pellets. For sawdust, the density was 120 kg/m^3^, whereas for shavings I and II, it was approximately 50 and 100 kg/m^3^, respectively. The highest bulk density of pellets was obtained for the most compacted material using cylindrical-shaped single particles, which were randomly oriented during filling. When an external pressure was exerted, the structure of the bed becomes more arranged. In the case of sawdust, the small size of single particles, as compared with the particles of shavings, determined the density value. In the case of materials with different moisture contents, an increase in the moisture content resulted in a higher density, probably owing to the combined effects of the action of water as a lubricant and the higher deformability of wet particles. The increase in the moisture content from 10% to 50% resulted in an average value of density increase in fragments. When the normal pressure was increased to the maximum value, the density of materials increased by approximately 10%, from 167 kg/m^3^ to 180 kg/m^3^. A comparison of the densities of the materials related to the application of various rotors shows that the highest density of 177 kg/m^3^ was obtained for the screw-like rotor shape. This was probably due to the easy sliding of tested materials over the slant surface of the rotor blades and easy consolidation of the samples. When the other rotors were used, the materials were suspended on the top of the rotors and sliding was not observed. The statistical analysis shows that the density of the examined materials was most strongly affected by the type of biomass for which the test function F was 757.96. The moisture content and normal pressure influenced the density in a weaker degree with F = 181.62 and F = 32.37, respectively. The least understandable and predictable factor was the rotor type, for which F = 7.11 influenced the density only in a small extent.

### 4.2. Coefficient of Sliding Friction, µ

**Figure 4:**
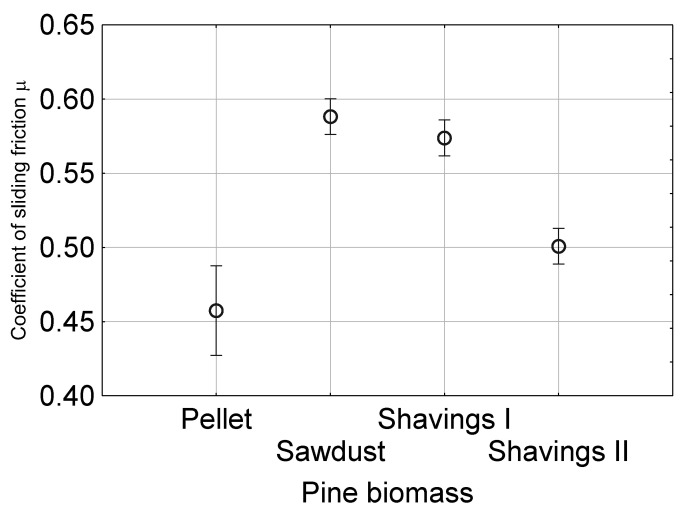
Mean value of the coefficient of sliding friction with 0.95 confidence intervals for different types of pine biomass and construction materials.

**Figure 5:**
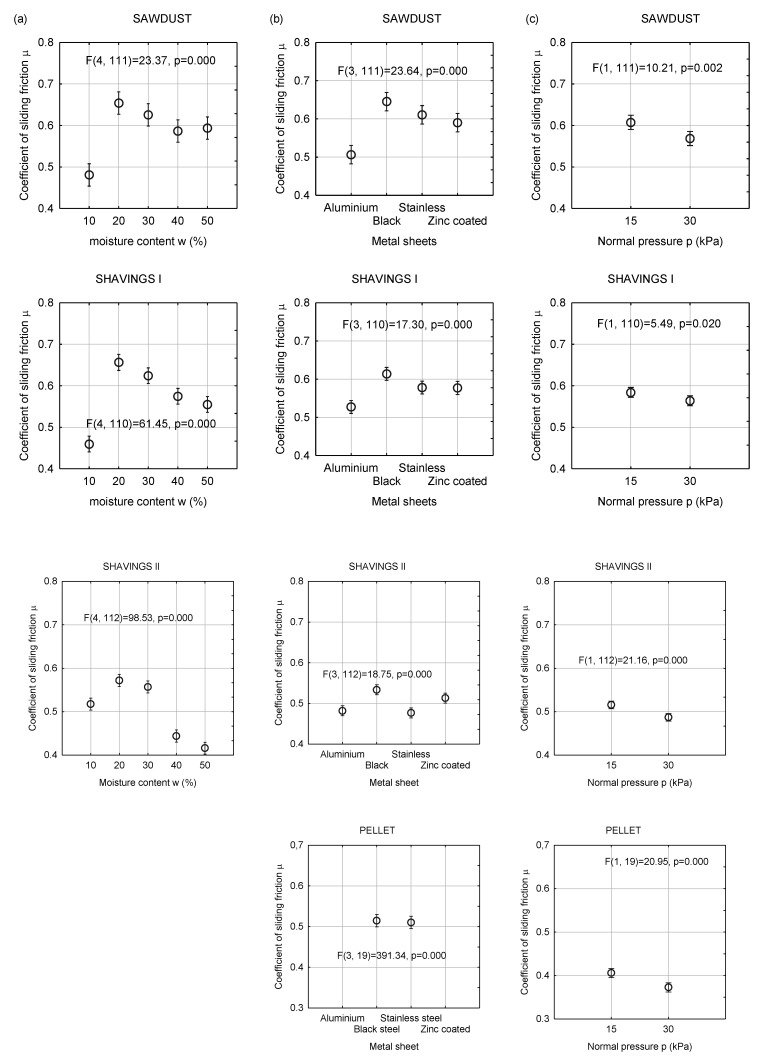
Mean value of sliding friction coefficient with 0.95 confidence intervals for various materials in dependence on moisture content (**a**), kind of sheet material (b) and normal pressure (c).

The coefficient of sliding friction between the layer of the granular and construction materials allow for the estimation of forces opposing movements in processing equipment. The determination of the coefficient of sliding friction is necessary for the design of devices in contact with granular biomass. Figure 4 and Figure 5 show the mean coefficient of the sliding friction measured for the tested biomass and construction materials and the relationships between the coefficient of sliding friction under various levels of normal pressure and moisture content

Significant differences between the coefficients of sliding friction for the examined materials were observed. The highest coefficients of sliding friction were obtained for the sawdust and shavings I, with values of 0.58 and 0.56, respectively. The coefficient of the sliding friction for shavings II was 0.5, and for pellet, it was the lowest at 0.45. Such large differences between the values of µ resulted from the size of individual particles; the particles of sawdust had the largest size. When the size decreased, the contact surface increased and resulted in an increase in the force needed to break the contact between particles and the surface of the construction materials. Sawdust has the smallest particles. Shavings I have larger individual grains as compared to sawdust, but its particles are easily deformed under external stress. Therefore, a high coefficient of sliding friction was also obtained for this material. In the case of shavings II, the particles are more elongated and thicker and thus less deformable under load. Moreover, the material remained in less contact with the construction surface because the load did not cause sufficient crushing on the surface through permanent structures built by the shavings II material. A similar phenomenon was observed in the case of pellets. The smooth surface of individual particles, additionally covered with lubricating layers created during the production of pellets, was an additional factor.

Figure 5 shows the relationships between the coefficient of sliding friction and the moisture content, type of construction materials and normal pressure. The effect of moisture content was not determined for pellets. In the case of other materials, an increase in the moisture content from 10% to 20% resulted in an increase in the coefficient of sliding friction. For the 20% moisture content, the highest mean values of µ were obtained for sawdust and shavings I (approximately 0.65 and 0.56, respectively). Further increase in the moisture content of the material reduced the coefficient of sliding friction. At a moisture content of 50%, the lowest coefficient of sliding friction was determined for shavings II (µ = 0.42) and the highest for sawdust II (µ = 0.60). For shavings II, the coefficient of sliding friction decreased from 0.65 to approximately 0.55. A further increase in the moisture content led to the occurrence of free water on the surface of the biomass material particles acting as a lubricant. Differences in the coefficient of sliding friction between the materials are a result of the interaction of the contact area between the biomass and the construction material and unit strength of the bond.

The lowest coefficients of sliding friction were obtained for the aluminum sheet: µ = 0.50 for sawdust, µ = 0.52 for shavings I, µ = 0.48 for shavings II, and µ = 0.27 for pellets. The lowest coefficient, i.e., 0.25, was found for pellets and galvanized steel. Depending on the type of biomass materials, the coefficient of sliding friction of stainless steel and galvanized steel varied from 0.25 to 0.62.

For the examined biomass materials, an increase in the normal pressure reduced the coefficient of sliding friction by approximately 5%. The probable reason for this effect was the increase in the amount of free water in the sample due to the consolidation.

### 4.3. Shear Behavior, a Torque of Resistance T

**Figure 6:**
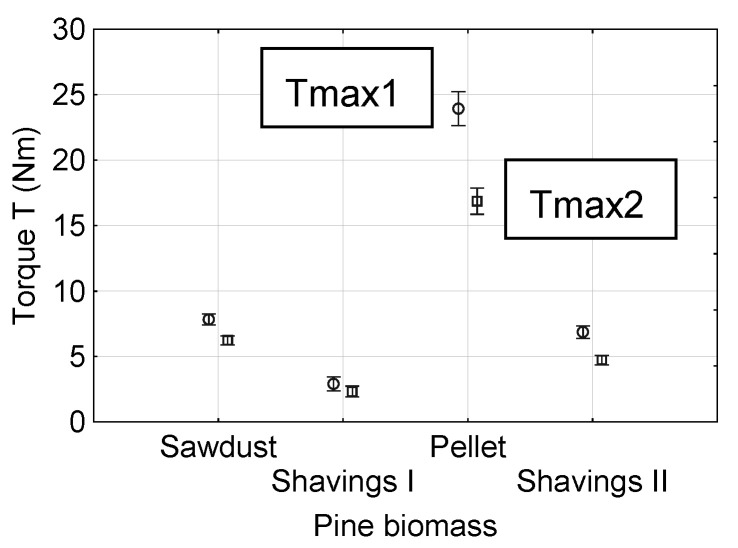
Mean value of the maximal shear torque with 0.95 confidence intervals for different types of biomass.

**Figure 7:**
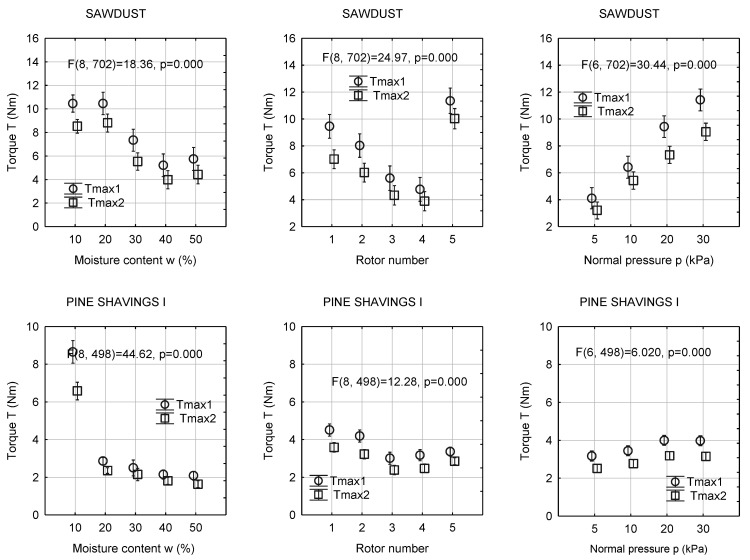

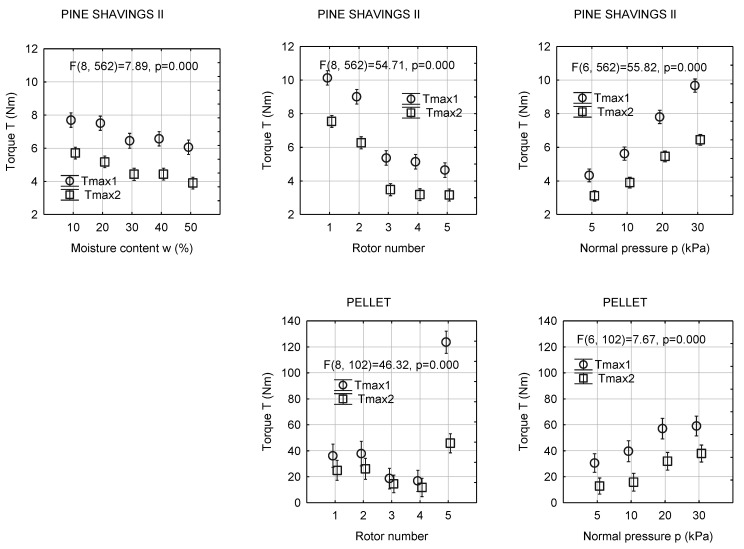
Mean value of maximal shear torques with 0.95 confidence intervals in dependence on moisture content, rotor number, and normal pressure.

Another objective of this study is to determine the shear resistance of the biomass against the rotation of the rotor measured as the value of torque for different moisture contents of the material. Accordingly, five types of rotors used in industrial practice and four levels of normal pressure were utilized in this study. The results of the tests are shown in Figure 6 and Figure 7. The highest values of the torque T were obtained for pellets, 25 and 16 Nm for the first maximum (Tmax1) and second maximum (Tmax2) after relaxation, respectively. For sawdust and shavings II, the torque values were approximately 25% smaller than those found for pellets, which were Tmax1 = 7 and Tmax2 = 6 Nm. The lowest values of Tmax1 and Tmax2, i.e., approximately 2.5 Nm, were found for shavings I.

For the examined materials, the torque values after the relaxation were lower than the first rotation torque values. The largest differences between the two torque values were found for pellets, where the structure of the material is stable until the first loss in strength. The rotor was then rotated in the space produced during the first rotation. For more homogeneous materials, i.e., sawdust and shavings I, the biomass returned to the initial structure or was closer to it. The values of torque, measured for shavings II, resulted from the thick elongated individual particles of this material.

The relationships between the value of the torque and moisture content, normal pressure, and rotor type are presented in Figure 7. The values of the torque decreased with the increasing moisture content for the examined materials. The highest decrease in T was obtained in the case of shavings I, where an increase in the moisture content from 10% to 20% resulted in a decrease in torque from approximately 8 Nm to approximately 2 Nm (i.e., 70%). In sawdust and shavings II, Tmax1 and Tmax2 decreased by approximately 50%. For these materials, in the entire range of moisture content, Tmax1 was approximately 2 Nm higher than Tmax1, and the difference was the highest among the examined materials.

The type of rotor used in the vane shear testing had a significant effect on the measured torque. The lowest values of T were found for rotors 3 and 4 for the tested materials. This is the result of the face area interacting with the material during the rotation of the rotor. In this case, the contact of the side surfaces is also limited. For sawdust and pellets, rotor 5 generated the highest value of torque. This is due to the contact surface with relatively smaller sawdust and pellet particles and the compaction of the material through the auger rotor. For the other biomass materials, the torques obtained with rotor 5 were compared with those determined for rotors 3 and 4.

In the case of more homogeneous materials, i.e., sawdust and pellets, rotors 1 and 2 generated slightly higher torques than rotors 3 and 4. This result is probably an effect of the larger contact area during shearing. For shavings II, the highest torque values were obtained for rotors 1 and 2, and they were approximately two times higher than those determined for other rotors. For shavings I and II, the values of T measured with rotor 5 were comparable with those determined for rotors 3 and 4. The increase in the normal pressure in the chamber resulted in an increase in the torque values for the biomass materials.

## 5. Discussion

In this article, we present the results of the examinations of the density, coefficient of sliding friction, and shear torque of pine granular materials popular in the market. The effects of the material type, moisture content, normal pressure, and type of shearing tool were studied. This paper reveals that the material disintegration and shape of the individual particles significantly affected the mechanical parameters determined in the applied tests. These results corroborate the findings reported earlier by Mattson and Kofman [11] and Littlefield [12] for willow shots and pecan shells, respectively. For the examined materials, differences between the determined parameters were determined. These were a result of the different contact conditions between the given granular biomass and surface of samples of construction materials or different shapes of vane tools (rotors). The results were also influenced by the shape of particles. In the case of elongated and irregular particles, the particles tended to aggregate, which led to the creation of larger assemblies that increased the bed strength.

In the case of density, the results are similar to those determined by authors using larger chambers and one type of rotor [18]. The results show that a reduction in the diameter of the chamber did not influence the values of parameters, and a smaller apparatus could be used for the determination of biomass properties. The highest values of bulk density were obtained for pellets, which corroborate previous results. The pellets had a high density owing to densification of wood material during pellet production. Slightly different results were obtained for the measuring chamber equipped with rotor 5. In this case, the differences resulted from the shape of the tool. Regardless of the type of biomass materials, density was the highest for rotor 5 because of the sliding of the biomass through the tool until the contact with the bottom. The results of the tests will help in the design of the dimensions of technological equipment using a given material.

The literature review has shown the lack of data regarding the sliding friction properties of various fractions of granular pine biomass. Existing studies on the sliding friction, presented in [1,3,8,21], did not address biomass originating from pine wood. Sliding friction testing on construction materials has been widespread for many years. In the case of pine biomass, data on sliding friction are important in the design of technological operations wherein these materials are involved. A study conducted by Zulfigar et al. [1] confirmed the decrease in the coefficient of sliding friction with an increase in pressure and the effect of various types of metal sheets on µ. Ghorbani et al. [22] examined alfalfa grinds similar to those presented in this project. The analysis of the effect of the size and moisture content of materials on the sliding and internal friction properties provided findings opposite to those presented in this study. The authors reported an increase in the friction with increasing moisture content and increasing particle size. The differences between the results obtained for alfalfa grinds and pine biomass may originate from the fact that alfalfa grinds are the chaffs of parts of green plants, which have a different structure and exhibit different behaviors. The state of water in these materials is also different. A similar tendency was observed by Larsson [21] for reed canary grass powder and by Mani et al. [23] for corn stover grind.

The results of the study on the impact of pressure on the sliding friction between pine biomass materials and construction materials are similar with those obtained by Craven et al. [24] for different types of wood biomass. These authors reported a decrease in the coefficient of sliding friction with the increase in pressure. These findings were confirmed by Mani et al. [23] and Larsson [21] for corn stover grind and reed canary grass, respectively. The tests carried out by Wu et al. [8] for wood pellets and wood chips showed approximately 10% lower values of the coefficient of sliding friction for pellets as compared with the coefficients of sliding friction determined for other materials. In the design of equipment for processing and transport of pine biomass, the minimal sliding friction is desirable. Such optimal conditions should be adopted. In the case of all the examined materials, the use of higher-value consolidations resulted in a reduction in the coefficient of sliding friction.

A simple version of the vane tester, previously used by Stasiak et al. [17] for determining the properties of pine woodchips, was used in this study to determine the torque values for pine biomass of various particle size distributions with different rotors. The literature review has shown that such investigations have not been conducted before. Knowledge on these parameters may be used to estimate loads when emptying a chamber with screw feeders, arch breakers, and mechanical bin actuators and in the process of pellet production, in which different driver shapes are used. In this study, only one speed of rotation was applied, because no effect of the rotational speed on the torque value was observed by Stasiak et al. [17,18] in their previous studies. The experimental results obtained for various biomass materials differed from one another and were strongly affected by the differences in the size and shape of individual particles of the examined materials. The tests conducted in the new apparatus for different rotors have confirmed that the values of torque are higher for materials composed of larger particles and for more heterogeneous materials.

The highest torque values were found for pellets. In the case of this material, the use of rotary conveyors should be limited during shredding. For the other three materials, the use of the carriers was justified. The higher moisture content of the material causes the decrease in the resistance values. The use of tools in technological processes with a shape similar to rotors 3 and 4 was considered most effective.

A method based on the vane shearing was developed for the rheological examinations of various materials [25,26,27,28]. The vane tester is a common and standardized device used for determining soil mechanical properties [29]. The explanation and evolution of the vane shear method is described in previous paper of Stasiak et al. [17]. The results of the present study confirm the efficiency of the use of vane shear tester in the determination of mechanical parameters of granular biomass.

## 6. Conclusions

The vane tester with different rotors could be used to estimate the loads acting on conveyors while emptying of the silos or to compare quickly the different lots of material.

The particle size of granular biomass affects its technological parameters. In this study, the highest value of bulk density was obtained for pellets. An increase in the moisture content and normal pressure resulted in an increase in the bulk density.

The size of biomass particles and type of construction materials were found to significantly affect the coefficient of sliding friction. The highest coefficients of sliding friction were obtained for sawdust and shavings I, whereas the lowest was obtained for pellets. The increase in the moisture content of the biomass from 10% to 20% increased the coefficient of sliding friction between the granular materials and construction materials. Further increase in the moisture content of the materials resulted in the reduction of the coefficient of sliding friction.

Regardless of the type of biomass material, the highest coefficients of sliding friction were obtained for the black steel sheet. The torque was determined by the type of biomass material and rotor shape. The highest values of the torque were obtained for pellets, and the torque decreased with increasing moisture content of the materials.

The type of rotor used in the vane shear testing had a significant effect on the measured torque. The lowest values of T were found for rotors 3 and 4 for the tested materials. These types of rotors seem to be most suitable for handling this type of biomass.

In the presented paper we limited investigations to pine biomass only. In the future, it would be advisable to determine the effect of the fragmentation of other types of plants used in the power industry.

## Figures and Tables

**Table 1:** Size distribution of the pine biomass materials (Stasiak et al., 2019).

Material	Particle Size (mm)	Fraction (%)	Material	Particle Size (mm)	Fraction (%)	Material	Particle Size (mm)	Fraction (%)	Material	Particle Size (mm)	Fraction (%)
Sawdust	<0.1 0.1–0.2 0.2–0.3 0.3–0.4 0.4–0.5 0.5–0.6 0.6–0.9 0.9–1.0 1.0–1.6 1.6–2.0 2.0–3.2 3.2–5.0 5.0>	8.70 21.60 14.00 13.80 6.50 6.40 9.80 12.60 0.40 2.10 3.90 0.20 0.00	Shavings I	<0.1 0.1–0.2 0.2–0.3 0.3–0.4 0.4–0.5 0.5–0.6 0.6–0.9 0.9–1.0 1.0–1.6 1.6–2.0 2.0–3.2 3.2–5.0 5.0>	0.51 0.00 0.00 0.35 0.32 0.43 1.01 5.69 0.23 2.39 12.53 8.71 67.84	Shavings II	<3 3.0–8.0 8.0–16.0 16.0–30.0 30.0–70.0 70>	2.50 2.50 1.30 22.32 63.64 7.74	Pellets ø 6 mm	3.0–8.0 8.0–16.0 16.0–30.0 30.0–40.0	11.00 54.62 29.88 4.50